# High frequency distribution of heterogeneous vancomycin resistant *Enterococcous faecium* (VRE*fm*) in Iranian hospitals

**DOI:** 10.1186/1746-1596-8-163

**Published:** 2013-10-02

**Authors:** Leili Shokoohizadeh, Ashraf Mohabati Mobarez, Mohammad Reza Zali, Reza Ranjbar, Masoud Alebouyeh, Türkan Sakinc, Liaqat Ali

**Affiliations:** 1 Department of Bacteriology, Faculty of Medical Sciences, Tarbiat Modares University, P. O. Box: 14115-111, Tehran, Iran; 2 Research Center of Gastroenterology and Liver Diseases Shahid Beheshti, University of Medical Sciences, Tehran, Iran; 3 Molecular Biology Research Center, Baqiyatallah, University of Medical Sciences of Tehran, Tehran, Iran; 4 Department of Internal Medicine II, University Hospital Freiburg, Freiburg, Germany; 5 Faculty of Biology, Albert Ludwigs University of Freiburg, Freiburg, Germany

**Keywords:** *E. faecium*, Vancomycin resistant, Hospital and PFGE

## Abstract

**Background:**

*Enterococcus faecium* is a multi-resistant nosocomial pathogen causing infection in debilitated patients**.** Vancomycin-resistant *enterococcus faecium* (VRE*fm*) are a major concern and increased dramatically worldwide especially in hospitals environment. The current study focused on determining the high prevalence and distribution patterns of antibiotic resistance and also its genetic linkages among various VRE*fm* strains isolated from indoor hospitalized patients in four major Iranian teaching hospitals of Tehran.

**Methods:**

The clinical samples were obtained from hospitalized patients during September 2010 to June 2011 from different teaching hospitals of Tehran. Antibiotics Resistance patterns, minimum inhibition concentration (MIC) value for vancomycin, ampicillin, gentamicin and presence of genetic linkage among the isolates were determined by pulsed-field gel electrophoresis (PFGE).

**Results:**

Overall, total of 92 (41.4%) isolates were identified as *E. faecium*, 45 (49%) were resistant to vancomycin with an MIC_50_ of ≥128 mg/L*.* The results showed that simultaneous resistance to teicoplanin, ampicillin, gentamicin, ciprofloxacine, tetracycline and erythromycin were observed the most frequent pattern. All the vancomycin resistant *E. faecium* isolates carried the *van*A gene. intensive care units (ICUs) and Kidney transplantation, are most probably the wards with highest risk of infection by VRE. 17 pulsotypes were also detected by PFGE, most of the related pulsotypes belongs to the same hospitals.

**Conclusions:**

This study shows the high alarming prevalence of *Enterococcus faecium* infection and similar clones of VRE*fm* strains in Iranian hospitals with threatening resistance phenotypes.

**Virtual slides:**

The virtual slides for this article can be found here: http://www.diagnosticpathology.diagnomx.eu/vs/1270863903102282

## Background

Enterococci have been regarded as low grade pathogens in the past, but in recent years it has rehabilitated into major pathogen in nosocomial infections [1]. Urinary tract and surgical site infections, bacteremia and endocarditis in immunocomprised patients are frequently caused by these bacteria in hospital [2]. The best therapy for enterococcal infection is the combination of aminoglycoside antibiotic (e.g. gentamicin) with a β-lactam antibiotic (e.g. ampicillin) or glycopeptides (e.g. vancomycin). However, vancomycin resistant enterococci (VRE) do not respond to these medications and thus there is limited option of treatment [3]. Although there is no report yet available of vancomycin resistant *E. faecium* (VREfm) outbreak in Iran but there is ample evidence showing that the frequency of these strains are increasing in hospitals and therefore caused a major therapeutic concern in recent years [4,5].

Characterization of VRE*fm* strains is crucial for the effective management of infection caused by this organism [6]. This characterization could be achieved with simple assays such as screening for resistance against antibiotics or more complex methods like Pulsed-field gel electrophoresis (PFGE) which is a powerful genotyping method used to study enterococcal distribution [7]. Routes of transmission and genetic linkage of these hospital isolates were examined in many epidemiological and molecular studies. Clonal spread of VRE*fm* strains has been associated with disease outbreak in hospitals [7]. However, different studies in Iran suggest the heterogeneity and polyclonal distribution of nosocomial infection in hospital environments [8-10].

Detection of antibiotic resistance patterns of VRE isolates against a vast spectrum of antibiotics and understanding how these strains transmitted based on their genetic features could be useful in eliminating the infection in hospital. Therefore, the main objective of this study to investigating high frequency prevalence and antibiotic resistance patterns of VRE*fm* isolates. We also analyzed the clonality and the genetic linkage between the strains by comparing their resistance patterns and pulsotypes in four major teaching hospitals in capital city Tehran, Iran.

## Materials and methods

### Identification of bacterial strains

The sampling was carried out in four major teaching hospitals in Tehran from September 2010 to June 2011. The isolates were obtained from clinical samples of indoor hospitalized patients (urine, wound, blood, abscess, sputum, bile, body fluid, intravenous catheter and trachea). All Enterococci isolates were identified according to their genus and species levels by gram staining, catalase reaction, growth in 6.5% NaCl, motility assessment, use of arabinose, bile and esculin hydrolysis and also pigment production after their growth on enterococcus selective agar (BBL, USA) all based on Falkman and Collins criteria [11]. PCR based study was conducted by using specific primer (*ddl*_E.faecium_) for each *E.faecium* species strains [12]. Protocols conformed to the ethical guidelines of the 1975 Declaration of Helsinki and were approved by Research Ethics Committee of the Tarbiat Modares University.

### DNA extraction and polymerase chain reaction

DNAs from different bacterial isolates were extracted by using appropriate DNA extraction Kit (Sinagene, Iran).

For PCR amplification assay, specific primers of *ddl*_E.faecium_ and vancomycin resistance genes (*van*A and *van*B) were used as describe previously [12]. *E. faecium* ATCC 51559 and *E. faecalis* ATCC 51229 as *van*A and *van*B standard encoding strains were used.

### Antimicrobial susceptibility test

Antimicrobial susceptibility test for isolates of *E. faecium* was performed against vancomycin (30 μg), teicoplanin (30 μg), gentamicin (120 μg), ampicillin (10 μg), erythromycin (15 μg), ciprofloxacin (5 μg), tetracycline (30 μg), chloramphenicol (30 μg), nitroforantoin (300 μg), quinopristin-dalfopristin (synercid) (15 μg) and linezolide (30 μg) (Mast, UK), by the disc diffusion method. Vancomycin, ampicillin and gentamicin MICs (Minimum inhibitory concentration) were determined by the agar dilution method. The results were interpreted according to the Clinical and Laboratory Standards Institute guidelines (CLSI- 2011).

### Pulsed-field gel electrophoresis

Genomic typing of isolates was performed by PFGE. Genomic DNA was prepared in low melting agarose plugs as described by Saifi *et al.*[8]. Restriction enzyme *Sma*I (Roche, Manheim, Germany) was used to digest the DNAs in small slices of the agarose plugs. The plugs were placed in 1% agarose (Invitrogen, USA) that was in 0.5% TBE and were electrophoresed with switch times ramped from 5 s to 35 s at 6 V with a run time of 23 hours at 14°C and an angel 120 in the Bio-Rad CHEF-DRIII system. *Salmonella cholerasuis* serotype Branderup H9812 was used as the molecular size marker. The gels were stained with ethidium bromide and photographed under ultraviolet light. The banding patterns were clustered by unweighted paired group (UPGMA) method by Gelcompar II software version 4.0. Interpretation was done by using the guidelines set out previously [13].

## Results

### Prevalence of *E. faecium* species

**Figure 1:**
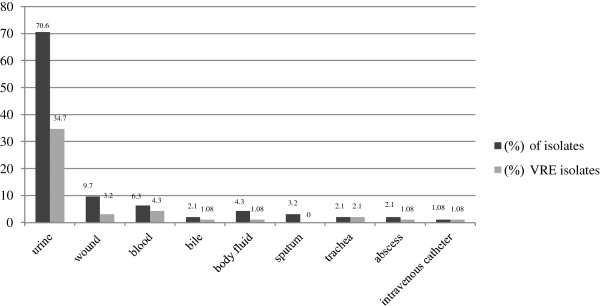
**The rate (%) of *Enterococcus faecium *strains isolated from clinical samples and (%) of VRE.**

A total of 222 enterococcal isolates samples were collected from clinical hospitalized patients. Overall, 41.4% (n = 92), 51.3% (n = 114) and 7.2% (n = 16) of the isolates were confirmed as *E. faecium*, *E. faecalis* and other species of enterococci respectively. Results of PCR for the *ddl* gene confirmed the biochemically identification in *E. faecium* isolates**.** Most of the clinical isolates belonged to urine 70.6% (n = 65) followed by wound samples 9.7% (n = 9) (Figure 1).

### Antimicrobial susceptibility testing

**Figure 2:**
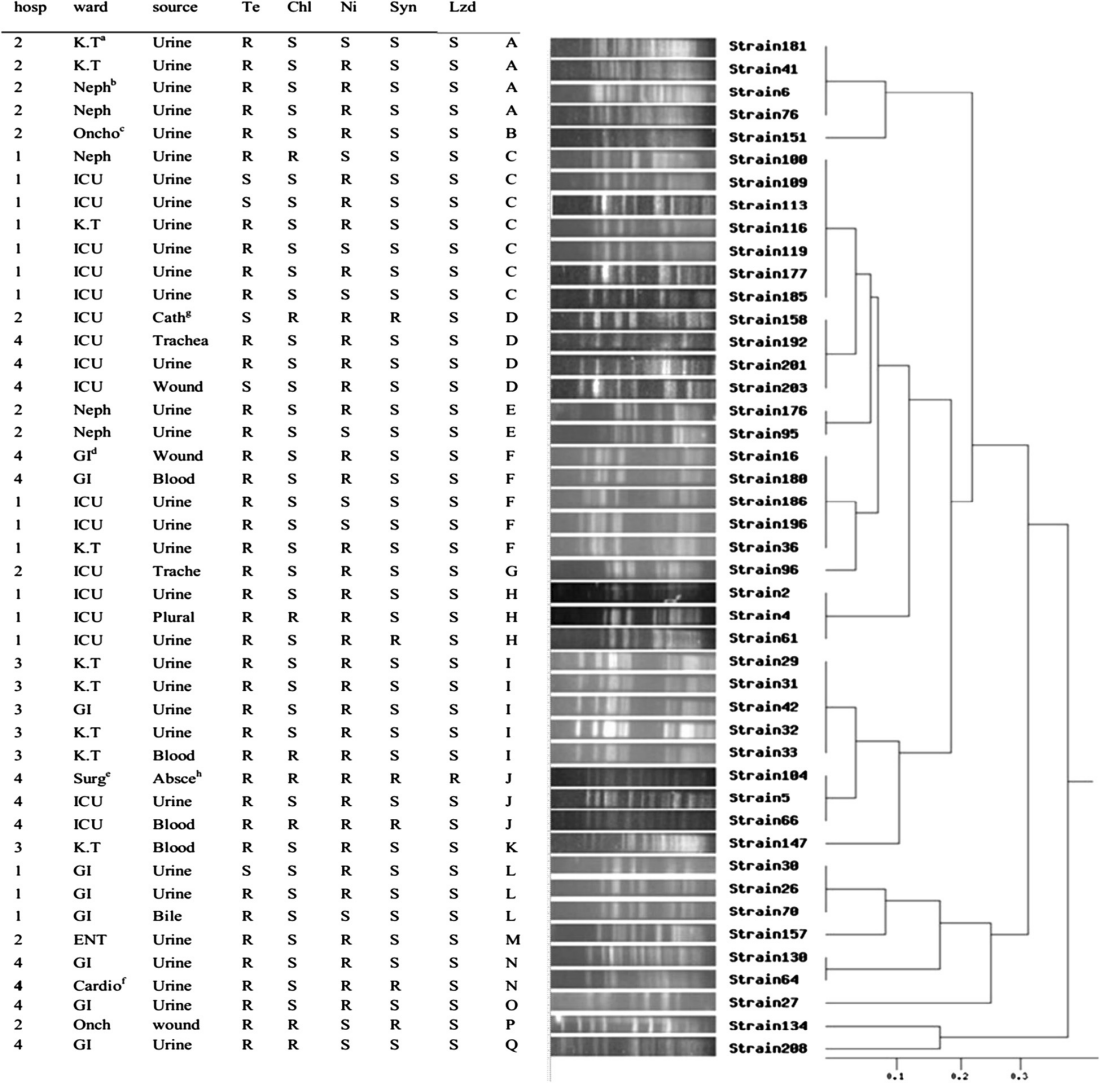
**Dendrogram cluster analysis of PFGE data for 45 VRE isolates with Tei/amp/Gm/Cip/E resistance phenotypes, based on hospitals, wards, source of infection, resistance to other antibiotics and pulsotypes a**; Gastroenterology, **b**; Kidney Transplant, **c**; Nephrology, **d**; Onchology **e**; Abscess, **h**; catheter, **g**; Cardiology, **f**; Surgery.

Antibiotic resistance analysis showed a high rate of vancomycin resistance 48.9% (n = 45) in the isolates under study. More (35.5%) VRE*fm* were isolated from hospitals by local code number 1. ICUs (n = 17, 38%) and kidney transplant wards (n = 9, 20%) have the larger number of these strains. All (100%) VRE isolates were also resistant to ampicillin, gentamicin, ciprofloxacin erythromycin and teicoplanin, this was followed by tetracycline (n = 36, 80%), nitroforantoin (n = 32, 71%), choleramphenicol (n = 8, 18%), quinopristin-dalfopristin (synercid) (n = 6, 13.3%) and linezolide (n = 1, 2%). MIC values for vancomycin, ampicillin and gentamicin were from 64 to 1024, 32 to 256 and 512 to 1024 μg/mL respectively. MIC_50_ for vancomycin and ampicillin was ≥128 mg/L and for gentamicin was ≥1024 mg/L. All VRE isolates harbored the *van*A gene. Resistance to vancomycin, ampicillin, gentamicin, ciprofloxacin, tetracycline, erythromycin and teicoplanin was the dominant antibiotic resistance phenotype (77.7%) (n = 35). All VRE isolates were selected for genotyping by PFGE (Figure 2).

### Genotyping

PFGE was performed in order to study the genetic linkage analysis among the clinical isolates. These isolates were divided into 17 pulsotype groups (A-Q) according to a similarity cutoff of ≥ 95%. The predominant pulsotype (C) comprised 7 isolates (15%). Six isolates (35.2%) showed unique PFGE pattern as shown in Figure 2.

## Discussion

Generally, *E. faecalis* has been known as the major cause of enterococcal infections, 10 times more prevalent than other enterococcus species. However, in recent years a remarkable change has seen from *E. faecalis* to *E. faecium* probably because of the emergence of VRE strains among members of this species in hospital environments [14]. The prevalence of *E. faecium* has been increased in Iranian hospitals during the last few years. In the present study, the ratio of infections due to *E. faecalis* to those caused by *E. faecium* was 1.2: 1 (51.3% versus 41.4%) which is still higher than some previous reports published from Iran and some other countries [4,15,16]. The increased ratio was supported by enhancement of *VREfm* strains*.* The emergence of *E. faecium* isolates with a high level of resistance to three main classes of antibiotics (i.e. glycopeptides, β-lactams and amino glycosides) against enterococci spp is a major concern in hospitals. Congruent with the results from the USA hospitals, all the studied VRE isolates in Iran are resistant to ampicillin, whereas European hospital-derived clones are reported to be vancomycin susceptible but resistant to ampicillin and gentamicin [17-19].

Our current study clearly shows that in Iran the frequency distribution of VRE is high compared to rest of the countries of the world [20–23]. Also, all of VRE isolates showed resistance to more than 6 antibiotics, 60% of VRE isolates showed threatening resistance phenotype to vancomycin, teicoplanin, ampicillin, gentamicin, ciprofloxacin, tetracycline, erythromycin, nitrofourantoin (Va/Tei/Am/Gm/Cip/Te/E//Ni). Moreover high MIC values (MIC_50_ ≥ 128) were also found for vancomycin and ampicillin as well as gentamicin MIC_50_ ≥ 1024.

It is also vibrant from the present study that all VRE isolates harbored vanA gene as describes previously. The wards related to kidney transplantation and nephrology, and ICU was estimated to be the ones with the highest risk of infection by VRE (Figure 2). The present study also reveals that linezolid and quinopristin- dalfopristin (synercid) were the most effective agents against the *E. faecium* isolates.

The dissemination of VRE*fm*, studies conducted in Iranian hospitals have found the dominancy in polyclonal among clinical isolates instead of clonal spreading as reported in USA and Europe but has consistency with Saudi Arabian hospitals [7,18-20].

The relative congruence of antibiotic resistance patterns and the specific PFGE patterns among the studied isolates demonstrates the presence of *E. faecium* strains with similar clone types in each of the hospitals e.g. pulsotypes C, H and L in hospital 1, pulsotype A in hospital 2, pulsotype I in hospital 3 and J in hospital 4 were identified.

The pulsotype patterns and resistance profiles suggest that there was an inter-hospital dissemination of pulsotype F and D (isolates were obtained from different sources and wards). Most of the other pulsotypes (e.g. Pulsotypes A, C and I) were related to the same hospitals. The best example for intra hospital dissemination was pulsotype C, because all of these strains were isolated from urine samples and from the same ward of a hospital.

## Conclusions

In conclusion, the increasing of polyclonal VRE*fm* with threatening resistance phenotypes is a serious concern in Iranian hospitals. High resistance to antibiotics in our study was most probably due to the selective antibiotic pressures. The highlighted results need a strong attention for surveillance programs to continuously monitor the nosocomial changes in bacterial resistance and the exchange of information about pathogens.

## Competing interests

The authors declare that they have no competing interests.

## Authors’ contributions

LS and AMM conceived the study. LS conducted the experiments and analyzed the results. LA and LS drafted the manuscript and made substantial contributions to the design of the study. LA, AMM, and TS critically reviewed the manuscript. MRZ, RR, TS and MA participated in data analysis. All the authors studied and approved the final manuscript.
